# Gelatin Methacryloyl–Riboflavin (GelMA–RF) Hydrogels for Bone Regeneration

**DOI:** 10.3390/ijms22041635

**Published:** 2021-02-06

**Authors:** Ryoma Goto, Eisaku Nishida, Shuichiro Kobayashi, Makoto Aino, Tasuku Ohno, Yuki Iwamura, Takeshi Kikuchi, Jun-ichiro Hayashi, Genta Yamamoto, Masaki Asakura, Akio Mitani

**Affiliations:** 1 Department of Periodontology, School of Dentistry, Aichi Gakuin University, 2-11 Suemori-dori, Chikusa-ku, Nagoya, Aichi 464-8651, Japan; ag173d09@dpc.agu.ac.jp (R.G.); kobaschu@dpc.agu.ac.jp (S.K.); i-makoto@dpc.agu.ac.jp (M.A.); tasuku@dpc.agu.ac.jp (T.O.); yukiwa@dpc.agu.ac.jp (Y.I.); tkikuchi@dpc.agu.ac.jp (T.K.); jun1row@dpc.agu.ac.jp (J.-i.H.); genta@dpc.agu.ac.jp (G.Y.); minita@dpc.agu.ac.jp (A.M.); 2 Department of Dental Materials Science, School of Dentistry, Aichi Gakuin University, 1-100 Kusumoto-cho, Chikusa-ku, Nagoya, Aichi 464-8650, Japan; masaki@dpc.agu.ac.jp

**Keywords:** GelMA, hydrogel, riboflavin, photocrosslinking, visible wavelength, osteoblast, tissue engineering

## Abstract

Gelatin methacryloyl (GelMA) is a versatile biomaterial that has been used in various biomedical fields. UV light is commonly used to photocrosslink such materials; however, its use has raised several biosafety concerns. We investigated the mechanical and biological properties of a visible-wavelength (VW)-light-crosslinked gelatin-based hydrogel to evaluate its viability as a scaffold for bone regeneration in bone-destructive disease treatment. Irgacure2959 or riboflavin was added as a photoinitiator to create GelMA solutions. GelMA solutions were poured into a mold and exposed to either UV or VW light. KUSA-A1 cell-laden GelMA hydrogels were crosslinked and then cultured. Mechanical characterization revealed that the stiffness range of GelMA–RF hydrogel was suitable for osteoblast differentiation. KUSA-A1 cells encapsulated in GelMA hydrogels photopolymerized with VW light displayed significantly higher cell viability than cells encapsulated in hydrogels photopolymerized with UV light. We also show that the expression of osteogenesis-related genes at a late stage of osteoblast differentiation in osteoblasts encapsulated in GelMA–RF hydrogel was markedly increased under osteoblast differentiation-inducing conditions. The GelMA–RF hydrogel served as an excellent scaffold for the encapsulation of osteoblasts. GelMA–RF hydrogel-encapsulated osteoblasts have the potential not only to help regenerate bone mass but also to treat complex bone defects associated with bone-destructive diseases such as periodontitis.

## 1. Introduction

The three key elements of tissue engineering are cells, growth factors, and scaffolds [1]. Tissue engineering involves growing cells in an appropriate three-dimensional (3D) environment, known as a scaffold, to which the cells attach and colonize. The major function of a scaffold is to replicate the structure of the natural extracellular matrix that assists the proliferation, differentiation, and biosynthesis of cells. In addition, a scaffold placed at the site of a defect to aid regeneration also prevents undesirable invading cells from occupying the defect’s space [2]. Hydrogels have been widely applied for biomedical purposes—including as scaffolds for regenerative medicine and as carriers for drug delivery—owing to their crosslinked network structure and have been modified in various ways to mimic the native extracellular matrix environment. Gelatin methacryloyl (GelMA), a photocrosslinkable material, is a useful, low-cost, and safe scaffold for tissue engineering and has been reported to promote cell proliferation, migration, and spreading in 3D environments [3]. GelMA hydrogel is obtained by incorporating methacrylate groups into the amine-containing side groups of gelatin, yielding a gelatin-based hydrogel [4]. The GelMA solution can form hydrogels that are irreversibly covalently crosslinked through exposure to ultraviolet (UV) light in the presence of a photoinitiator [3].

The construction of biomimetic bone microstructures through bone tissue engineering can promote the alignment and differentiation of cells in 3D environments. GelMA hydrogel provides a microenvironment that promotes the osteogenic differentiation of adult stem cells [5]. McBeth et al. demonstrated that GelMA hydrogel is able to trigger mineral deposition in primary normal human osteoblasts in the absence of any exogenous osteogenic factors [6]. The radical polymerization of GelMA hydrogel is generally initiated by exposure to UV light in the presence of a photoinitiator [7]. However, cell/tissue damage, including DNA damage, accelerated tissue aging, and cancer, can be caused by the UV light exposure [4] needed for GelMA photocrosslinking [8]. In addition, human osteoblasts are less resistant to UV exposure [9]. Thus, we focused on using visible-wavelength (VW) light with riboflavin [10] as a photosensitizer for the polymerization of GelMA hydrogels to avoid damaging osteoblasts with UV light.

When envisioning the use of GelMA in clinical regenerative treatment, the photoinitiator must also be a safe and applicable material for the human body. Riboflavin, also known as vitamin B2, is reduced by light, releasing free radicals, and is often used as a photoinitiator for the radical polymerization of hydrogels such as polyacrylamide gels [11]. Thus, riboflavin may be useful as a photoinitiator in the radical polymerization of GelMA hydrogel. In addition, riboflavin has also been considered for use in several biomedical applications [12,13,14], and the absorption spectrum of a riboflavin solution exhibits maximal absorbance at 222, 266, and 373 nm in the UV range as well as at 445 nm in the visible-light range [15]. Therefore, in the case of applying the GelMA hydrogel to defective or damaged organs for regenerative therapy, riboflavin may be useful as a possible photoinitiator of GelMA polymerization with irradiation with visible light instead of UV light.

The aim of our study was to reveal the most efficacious and safest environment for transplanting osteoblasts into bone defects. We investigated the in vitro feasibility of a novel method using a GelMA–riboflavin (GelMA–RF) hydrogel as a scaffold for bone regeneration based on osteoblast differentiation.

## 2. Results

### 2.1. Physical and Mechanical Properties of GelMA–RF Hydrogels

**Figure 1:**
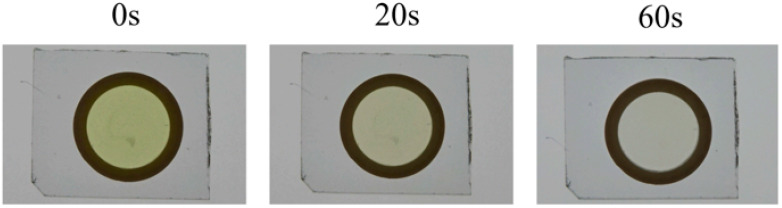
Gelatin methacryloyl–riboflavin (GelMA-RF) before and after photopolymerization. The three images show a 20% GelMA-RF hydrogel before and after photopolymerization under visible-wavelength (VW) light. Before photopolymerization, the GelMA–RF hydrogel was translucent and dark yellow. After 20 s of photopolymerization, the GelMA–RF hydrogel was translucent and pale yellow. After 60 s of photopolymerization, the GelMA–RF hydrogel was almost completely transparent. Furthermore, 10% and 15% (*w*/*v*) GelMA–RF gels were too weak to be handled for testing after 20 s and 60 s of photopolymerization (data not shown).

**Figure 2:**
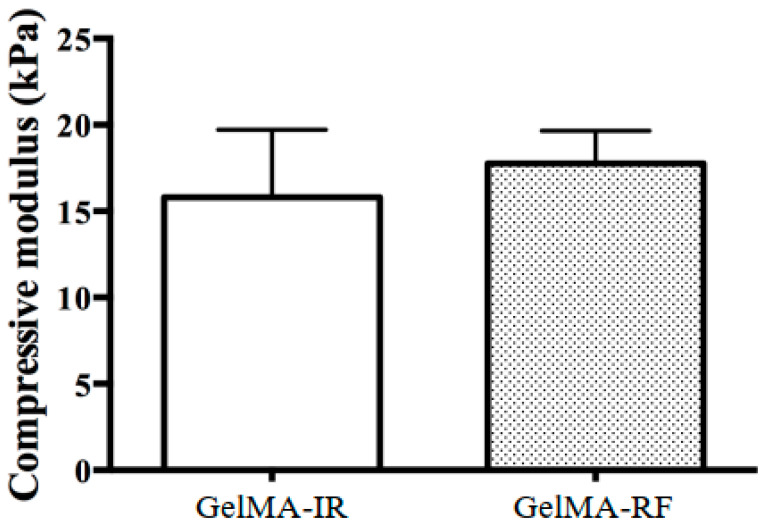
Mechanical properties of a GelMA–RF hydrogel and a GelMA–Irgacure2959 (IR) hydrogel. The compressive moduli of a GelMA-RF hydrogel and a GelMA-IR hydrogel were measured and appeared to not be significantly different. The stiffness of the two hydrogels was within the range suitable for osteoblast differentiation. Error bars represent the SD of measurements performed on five samples.

Figure 1 shows the morphology of a 20% (*w*/*v*) GelMA–RF hydrogel after photopolymerization under VW irradiation. A 20% GelMA–RF hydrogel disc irradiated for 60 s was colorless and completely transparent, while a similar disc irradiated for 20 s was translucent yellow, which indicated that unpolymerized RF remained. The gel exposed for 20 s could not be analyzed for mechanical properties, because it could not maintain its morphology outside the mold. In addition, 10% and 15% (*w*/*v*) GelMA–RF hydrogels were also difficult to remove from the mold because of unpolymerized RF and could not be analyzed for their mechanical properties. The 20% (*w*/*v*) GelMA–RF hydrogel disc irradiated with VW for 60 s and the 20% (*w*/*v*) GelMA–Irgacure2959 (IR) disc irradiated for 20 s with UV light showed similar elasticity, with compressive moduli of approximately 16 kPa and 17 kPa, respectively (Figure 2).

### 2.2. Cell Viability Following VW and UV Light Photopolymerization

**Figure 3:**
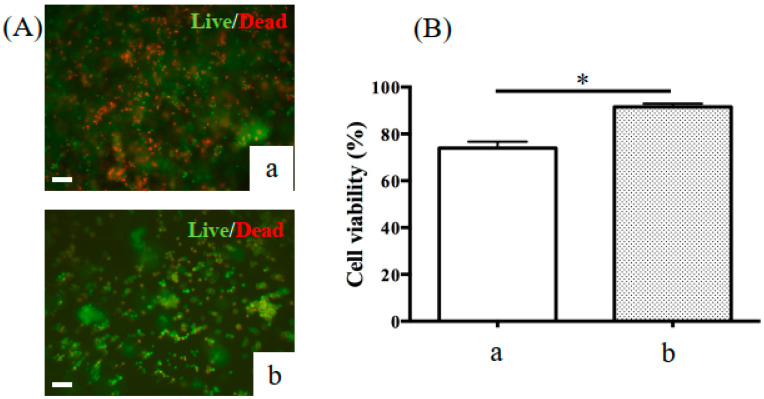
Representative fluorescence images of KUSA-A1 cells stained green (live) and red (dead). (**A**) The viability of cells encapsulated in 20% GelMA hydrogels after exposure to (**a**) UV light or (**b**) VW light and cultured in vitro for 1 day was measured. (**B**) Cell viability decreased when KUSA-A1 cells were exposed to (**a**) UV light as opposed to (**b**) VW light; * *p* < 0.05. UV: ultraviolet. Scale bars: 100 μm.

Figure 3 shows the viability of KUSA-A1 cells encapsulated in GelMA hydrogels photopolymerized with VW light for 60 s and UV light for 20 s and cultured in vitro for 1 day. KUSA-A1 cells encapsulated in GelMA hydrogels photopolymerized with VW light displayed significantly higher cell viability than those encapsulated in GelMA hydrogels photopolymerized with UV light. Therefore, the use of VW light for GelMA hydrogel photopolymerization prevented cell damage compared with the use of UV light.

### 2.3. Osteoblastic Differentiation of KUSA-A1 Cells in a 2D Environment

**Figure 4:**
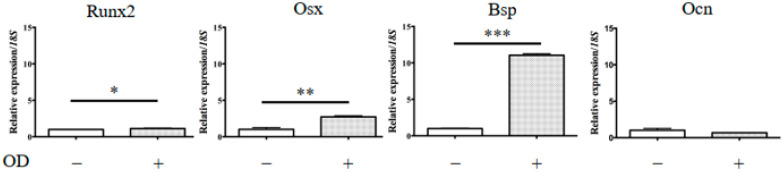
mRNA expression of osteogenesis-related genes in KUSA-A1 cells on a plastic plate. After 7 days of culture, the mRNA expression of the *BSP* gene in a 2D+ environment was markedly increased compared with that in a 2D environment. The data represent the mean ± SD of four independent experiments. Each experiment was performed in triplicate; * *p* < 0.05, ** *p* < 0.01, and *** *p* < 0.001. 2D: KUSA-A1 cells were cultured without osteogenic differentiation medium on a plastic plate; 2D+: KUSA-A1 cells were cultured with osteogenic differentiation medium on a plastic plate; Runx2: Runt-related transcription factor 2, Osx: osterix, Bsp: bone sialoprotein, Ocn: osteocalcin.

**Figure A1:**
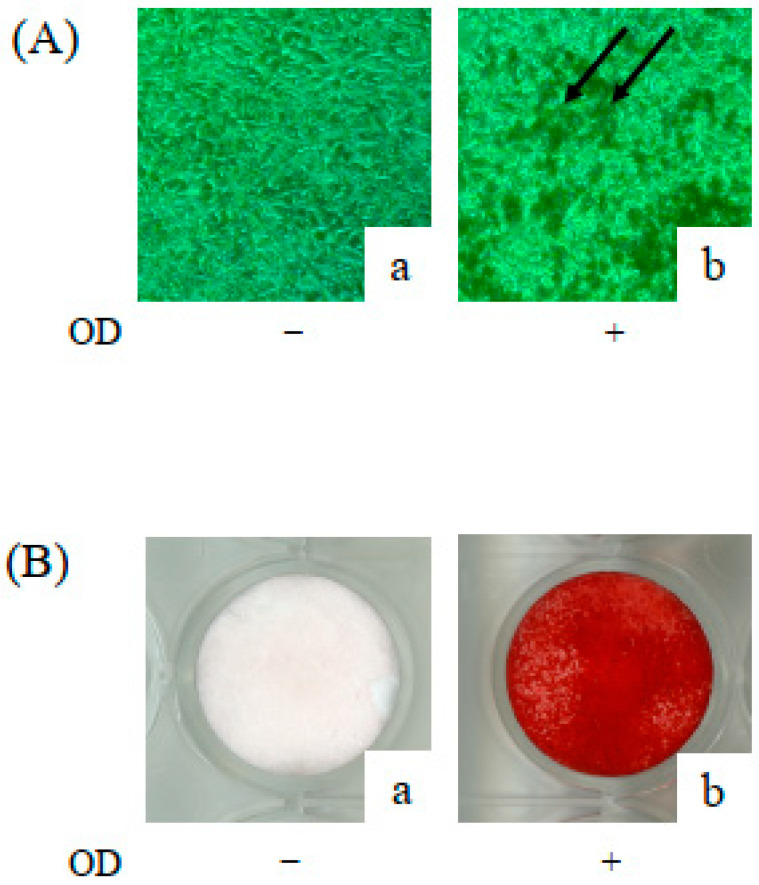
KUSA-A1 cells provide a suitable model of osteogenesis. Cells were cultured for 7 days without (−) or with (+) L-ascorbic acid phosphate, dexamethasone, and beta-glycerophosphate. (**A**) Mineralized nodules were observed (**b**, arrows). (**B**) Calcium deposition was visualized by alizarin red S staining (**b**). Mineral deposition by KUSA-A1 cells demonstrated that they have the capacity to differentiate into osteoblasts. OD: osteo-differentiation.

KUSA-A1 cells were treated with osteogenic differentiation supplements and then cultured for 7 days. Calcium deposited in the cells was then determined using alizarin red staining (Appendix A Figure A1). The levels of calcium deposition were dramatically increased in KUSA-A1 cells after 7 days. The relative expression of marker genes for the osteoblastic lineage in KUSA-A1 cells after osteogenic induction was investigated by qPCR analysis. It was found that KUSA-A1 cells osteo-induced for 7 days expressed bone sialoprotein (BSP) mRNA strongly and significantly compared with non-induced cells (Figure 4).

### 2.4. Expression of Osteogenesis-Related Genes in Osteogenic Induced KUSA-A1 Cells Encapsulated in GelMA

**Figure 5:**
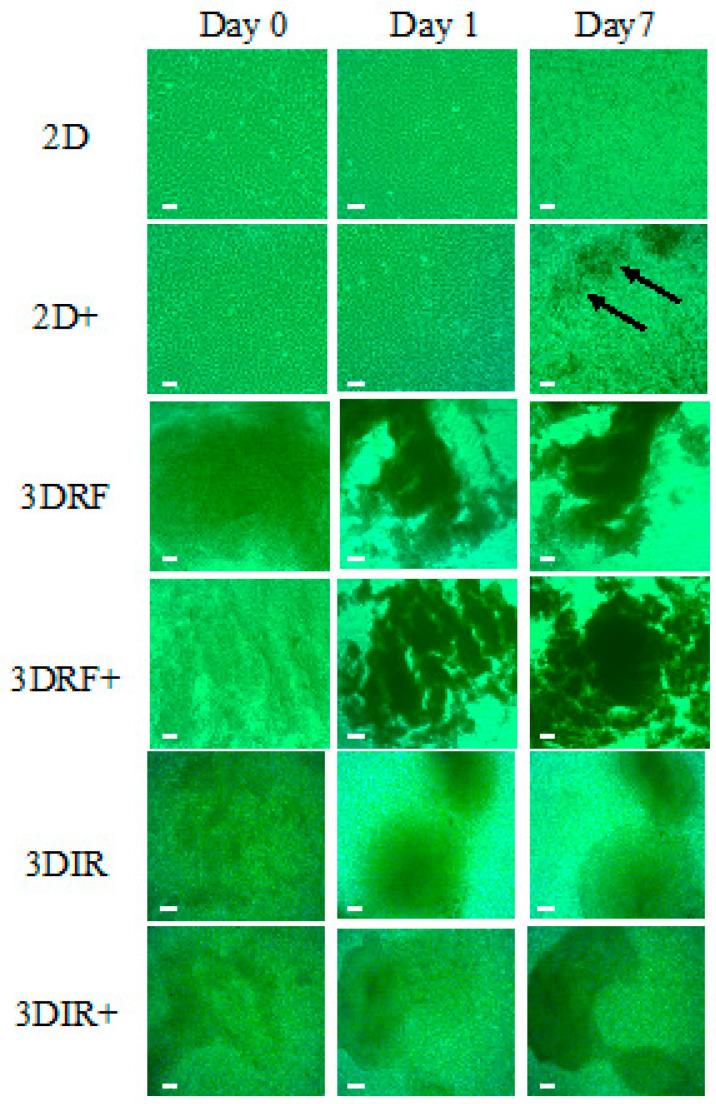
Cell morphology observations. 2D: KUSA-A1 cells were cultured without osteogenic differentiation medium on a plastic plate. 2D+: KUSA-A1 cells were cultured with osteogenic differentiation medium on a plastic plate. Discrete mineralized nodules are indicated by arrows. 3DRF: KUSA-A1 cells were encapsulated in GelMA–RF hydrogel without osteogenic differentiation medium. 3DRF+: KUSA-A1 cells were encapsulated in GelMA–RF hydrogel with osteogenic differentiation medium. 3DIR: KUSA-A1 cells were encapsulated in GelMA–IR hydrogel without osteogenic differentiation medium. 3DIR+: KUSA-A1 cells were encapsulated in GelMA–IR hydrogel with osteogenic differentiation medium. KUSA-A1 cells aggregated and exhibited spheroid-like formations in the GelMA-RF and GelMA–IR hydrogel cultures. Scale bars: 100 μm.

Figure 5 shows the significant effect of the GelMA–RF hydrogel or the GelMA–IR hydrogel on KUSA-A1 cell morphology. After 7 days of culture, mineralized nodules were observed in KUSA-A1 cells cultured with osteogenic differentiation medium on plastic plates (Figure 5, arrows). In contrast, aggregation and spheroid-like formations were observed for KUSA-A1 cells cultured within the GelMA–RF and GelMA–IR hydrogels (Figure 5).

**Figure 6:**
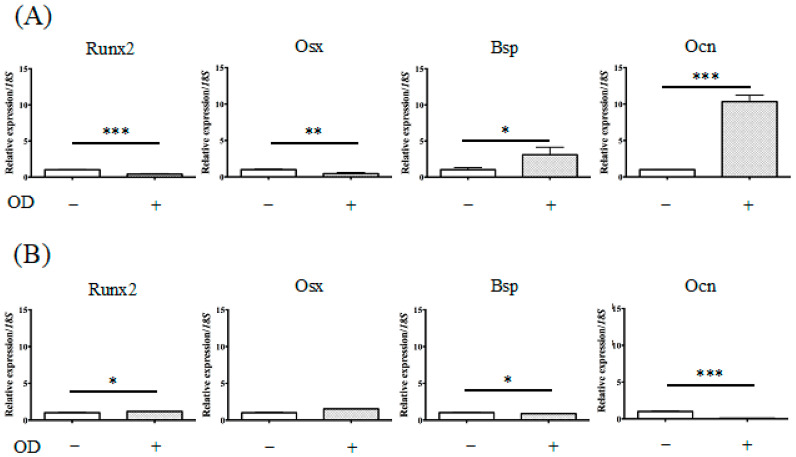
mRNA expression of osteogenesis-related genes in KUSA-A1 cells encapsulated in GelMA–RF hydrogel or GelMAIR hydrogel. (**A**) After 7 days of culture in a 3DRF+ environment, the mRNA expression of *Ocn* was markedly increased compared with that in the 3DRF environment. (**B**) Runx2 mRNA was significantly increased in 3DIR+ compared with that in the 3DIR environment. These data represent the mean ± SD of four independent experiments. Each experiment was performed in triplicate; * *p* < 0.05, ** *p* < 0.01, and *** *p* < 0.001. 3DRF: KUSA-A1 cells were encapsulated in GelMA-RF hydrogel without osteogenic differentiation medium.

Figure 4 and Figure 6 show the mRNA transcript levels of osteogenesis-related genes in KUSA-A1 cells cultured in 2D (plastic plate), 3DRF (GelMA–RF hydrogel), or 3DIR (GelMA–IR hydrogel) with or without osteogenic differentiation medium for 7 days. The mRNA expression level of osteocalcin (Ocn), which is expressed in a late stage of osteoblast differentiation, was markedly upregulated in 3DRF, while Runt-related transcription factor (Runx2) and osterix (Osx), expressed in an early stage of osteoblast differentiation, were downregulated in 3DRF (Figure 6A). Runx2 mRNA was significantly increased in KUSA-A1 cells encapsulated in GelMA–IR hydrogel with osteo-differentiation medium (Figure 6B). These results indicated the upregulation of osteogenesis-related genes for late-stage osteoblast differentiation in KUSA-A1 cells encapsulated in the GelMA–RF hydrogel and therefore that the 3DRF environment of the GelMA hydrogel may accelerate osteoblast differentiation.

## 3. Discussion

This study demonstrates the potential to achieve osteo-differentiation in GelMA–RF hydrogel culture systems without UV light exposure. We investigated the effect of VW irradiation on KUSA-A1 cells encapsulated in GelMA hydrogels using RF, and to the best of our knowledge, this is the first study to examine the use of osteoblast-laden GelMA–RF hydrogels for bone regeneration.

As previously stated, most reports of GelMA hydrogels have used UV light for photoinitiated radical polymerization. However, VW light is less damaging to cells than UV light [16]. Figure 3 shows that VW polymerization allowed better cell viability than UV polymerization. In bone regeneration in actual surgical practice, UV irradiation may affect not only osteoblastic cells in the scaffold, but also cells in tissues around the bone defect area. Changing the light source for photopolymerization from UV light to less harmful VW light would ensure greater safety in the clinical application of GelMA hydrogels.

One of our points of focus was the light irradiation conditions of GelMA–RF polymerization for obtaining physical characteristics similar to those achieved when using the conventional polymerization method. It took 60 s for unreacted riboflavin to disappear, as shown in Figure 1, and the compressive module of GelMA under this irradiation condition was equivalent to that obtained when using the conventional method. Because the cross-linked structure of GelMA–RF is considered to be similar to that of GelMA–IR, the compressive moduli of the two hydrogels should be the same if the concentration of GelMA is the same. Therefore, this means that GelMA–RF was sufficiently polymerized with an irradiation time of 60 s. In fact, when the irradiation time was 20 s, the gel could not be sufficiently cured, and its morphology could not be maintained outside the mold.

As shown in Figure 6, the compressive modulus of the GelMA–RF used in this study was suitable for 3D culture, but polymerization under other conditions was not investigated in this study. The size and thickness of the prepared gel were modeled in anticipation of an in vivo rodent study, and if they are changed, the appropriate polymerization conditions will also change. In order to use GelMA–RF in various fields, it is necessary to carry out research to evaluate the physical properties of GelMA by changing parameters such as riboflavin concentration, irradiation time, and irradiation intensity.

Monteiro et al. reported GelMA polymerization using lithium acylphosphonate as a photoinitiator and a visible wavelength dental curing light in an effort to procedures in dental surgery. The viability of the odontoblast-like cells was unchanged compared with that of cells polymerized by UV light; however, sufficient viability was measured [17]. In our study, we used KUSA-A1 osteoblasts, a murine bone marrow-derived mesenchymal stem cell line that is highly oriented towards osteocytic differentiation [18]. Huang et al. reported that Runx2 and Osx were highly expressed by osteoblasts in the early stage of osteoblast differentiation [19]. In contrast, Bsp and Ocn were regarded as late-stage markers of bone formation [19]. We observed different tendencies in the expression of ostetoblast differentiation markers in 2D culture and 3D culture after 7 days (Figure 4 and Figure 6). Notably, *Ocn* mRNA was particularly highly expressed in KUSA-A1 cells encapsulated in GelMA–RF hydrogel with osteo-differentiation medium (Figure 6). Interestingly, the mRNA level of *Runx2* and *Osx* was downregulated in KUSA-A1 cells encapsulated in GelMA–RF hydrogel with osteo-differentiation medium. In addition, KUSA-A1 cells aggregated and exhibited spheroid-like formations when cultured in GelMA–RF hydrogel (Figure 5). These results indicate that the cells cultured in the 3D environment had already shifted to the late stage of differentiation, suggesting that the 3D matrix structure of GelMA–RF hydrogel led to strong differentiation and maturation. GelMAy-RF hydrogel cultures appear to be suitable for osteogenesis by osteoblast cells in vitro.

Mesenchymal stem cells (MSCs) are the best-studied cells for bone regenerative therapy. In particular, bone marrow-derived MSCs have attracted considerable scientific and medical attention as an effective tissue engineering cell therapy [20]. MSCs have a progenitor cell population capable of giving rise to three mesodermal lineages: chondrocytes, osteoblasts, and adipocytes [21]. The MSC lineage fate depends on cell sensitivity to tissue elasticity. Engler et al. demonstrated that MSCs differentiated into chondrocytes, osteoblasts, and adipocytes on gels of appropriate stiffness [22]. On a 25–40 kPa-stiff gel, MSC expressed osteogenic markers (MyoD, osteocalcin, Cbfa1) [22]. In addition, MSCs were found to mature and differentiate into osteoblasts within a hard substrate of 11–30 kPa of stiffness after 7 days of culture [23]. These reports indicate that matrix elasticity directs cell lineage specification. The compressive modulus of elasticity of 20% GelMA–RF hydrogels was approximately 16 kPa (n = 5) (Figure 2), which falls within the range of appropriate stiffness mentioned above. Therefore, we believe that the matrix elasticity of 20% GelMA–RF is suitable for osteoblastic cells differentiation.

This study has several limitations. First, the photopolymerized conditions of GelMA–RF were determined according to the conventional polymerization method. Therefore, additional validation of riboflavin concentration and VW irradiation time and irradiation intensity would be valuable to confirm the conditions for osteoblast differentiation in GelMA–RF hydrogel. Second, only the compressive modulus was measured when examining GelMA hydrogel morphology. As an additional study of GelMA hydrogel morphology, further investigation of its physical and mechanical properties (i.e., by scanning electron microscopy (SEM) and studies of its degradability and swelling behavior) is needed to determine the suitability of GelMA–RF hydrogel for osteoblast differentiation.

Bone regeneration requires viable and proliferative cell sources that show osteogenic differentiation [24]. A number of bone tissue engineering approaches are being investigated, in which osteogenic cells are combined with scaffold materials. Osteogenic cells are responsible for the synthesis, organization, and remodeling of the new bone tissue. Scaffold materials are structural and logistical templates that must be combined with osteogenic cells and growth factors to achieve cell attachment and tissue development [25]. Hydrogels are one category of scaffold that have been widely used in tissue engineering and regenerative medicine [26]. One of the most important advantages of GelMA hydrogels is their compatibility with microfabrication techniques that could lead to the development of stem cell niches [27]. Hence, photocrosslinkable GelMA hydrogels have gained significant attention in the tissue engineering field [28]. GelMA–RF hydrogel could potentially be a useful graft material for bone tissue regeneration. Moreover, the use of GelMA–RF hydrogel, which can be induced to support osteogenic differentiation in vitro, may contribute to the development of novel periodontal tissue regeneration cell therapies and transplantation therapies for bone regeneration.

## 4. Materials and Methods

### 4.1. GelMA Hydrogel Preparation

**Figure 7:**
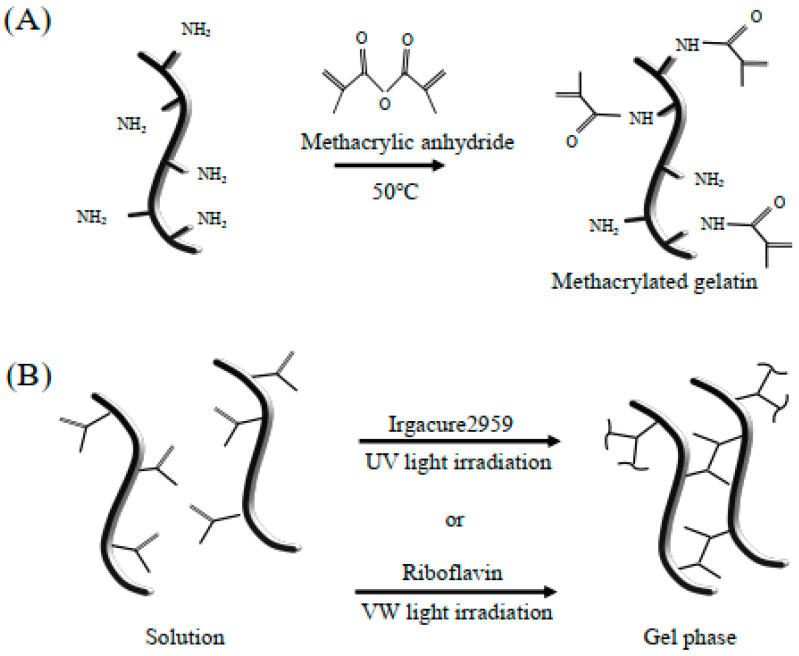
Synthesis of methacrylated-gelatin hydrogel. (**A**) Gelatin macromers containing primary amine groups were reacted with methacrylic anhydride to add methacrylate pendant groups. (**B**) To create a hydrogel network, the methacrylated gelatin was crosslinked using UV light irradiation in the presence of a photoinitiator, i.e., Irgacure 2959, or VW light irradiation in the presence of the photoinitiators riboflavin and triethanolamine. The solution was free flowing before UV or VW light treatment, and gelation occurred after crosslinking.

GelMA hydrogel was synthesized as described previously [3]. Briefly, type A gelatin from porcine skin was dissolved at 10% (*w*/*v*) in Dulbecco’s phosphate buffered saline (DPBS, Sigma-Aldrich, Madison, WI, USA) at 60 °C by stirring. Methacrylic anhydride (MA) was added to the gelatin solution at a rate of 0.5 mL/min while stirring at 50 °C, until the target volume was reached, and then the mixture was allowed to react for 1 h (Figure 7A). Following a 5× dilution with additional warm (40 °C) DPBS to stop the reaction, the mixture was dialyzed against distilled water using 12–14 kDa-cut off dialysis tubing for 1 week at 40 °C to remove the salts and methacrylic acid. The solution was lyophilized for 1 week to generate a white porous foam and stored at room temperature until further use [29].

GelMA was fully dissolved in Dulbecco’s modified Eagles medium (DMEM) (Sigma-Aldrich, Madison, WI, USA), then 4 mM IR (Irgacure2959; Sigma-Aldrich, Madison, WI, USA) [30] or 4 mM RF (riboflavin; Sigma-Aldrich, Madison, WI, USA) and 4 M triethanolamine (Sigma-Aldrich, Madison, WI, USA) as a coinitiator [31] was added to create a 20% (*w*/*v*) GelMA solution at 37 °C (Figure 7B).

### 4.2. Determination of the Polymerization Conditions of GelMA Hydrogels

The GelMA–RF solution (20 μL, 20% (*w*/*v*)) was poured into a silicone mold with controlled dimensions (⌀ = 8 mm, h = 0.8 mm) and then exposed to VW light (395–480 nm, VALO curing LED light, ULTRADENT JAPAN, Tokyo, Japan) for 0, 20, and 60 s. The Xtra power mode of the light source at 0.5 cm working distance was used for VW polymerization. IR has a UV absorption band at 276 nm and has minimal absorption at 365 nm [32]. The GelMA–IR solution was photocrosslinked by exposure to UV irradiation (365 nm, UV-LED light source, Hayashi-repic Co., Tokyo, Japan) in the silicone mold described above. UV irradiation working distance and time were 8.5 cm and 20 s [29]. The light irradiance of UV irradiation at a distance of 8.5 cm was 89 mW/cm^2^, and VW light irradiation at a distance of 0.5 cm was 1124 mW/cm^2^.

### 4.3. Mechanical Stability

The compressive modulus of elasticity of the GelMA hydrogels was measured using a mechanical testing machine (Instron 5980, Illinois Tool Works Inc., Norwood, MA, USA) in the absence of cells. The force was applied at a rate of 20% strain/min.

The compressive modulus was determined from the slope of the linear region corresponding to 5–10% strain. Change in the compression rate did not affect the compressive modulus values, indicating that the moduli were in the elastic region of deformation.

### 4.4. Cell Culture and Osteo-Induction in a Two-Dimensional (2D) Environment

The KUSA-A1 cell line was provided by Riken Cell BRC Cell Bank (Tsukuba, Japan) and maintained in DMEM supplemented with 10% FBS and 1% penicillin/streptomycin. The cells were subsequently cultured on a two-dimensional (2D) plastic plate in a 5% CO_2_ atmosphere at 37 °C. The cell culture medium was changed every 2 days. For osteogenic induction, cells were cultured until confluent, and then L-ascorbic acid phosphate, dexamethasone, and beta-glycerophosphate (Sigma, St. Louis, MO, USA) were added every other day for osteogenic differentiation. Calcium accumulation was detected by staining the preparations with a 1% alizarin red solution for 5 min [33].

### 4.5. Cell Encapsulation and Cell Viability in a 3D Environment

The 20% (*w*/*v*) GelMA was dissolved into DMEM (Sigma-Aldrich, Madison, WI, USA) at 60 °C by stirring. Then, 4 mM IR or 4 mM RF was added to create a GelMA solution. KUSA-A1 cell pellets (3 × 10^5^ cells) were resuspended in 20 μL of GelMA–IR solution or GelMA–RF solution, poured into a silicone mold, and photocrosslinked via exposure to UV light for 20 s or VW light for 60 s. After crosslinking, each cell-laden GelMA–IR hydrogel or GelMA–RF hydrogel was transferred into DMEM. Live cells were identified using a live/dead assay kit (Thermo Fisher Scientific, Yokohama, Japan). The live and dead cells were counted using ImageJ software (NIH, Bethesda, MD, USA) based on at least 3 locations in triplicate samples after 1 day of culture. The percentage of viable cells was then calculated based on the number of live cells relative to the total number of cells (live and dead) in the construct.

### 4.6. Cell Encapsulation and Osteo-Induction in a 3D Environment

Each cell pellet was resuspended in GelMA–RF or GelMA–IR solution, poured onto a cell culture insert (0.4 μm pore diameter; BD, Franklin Lakes, NJ, USA), and photocrosslinked by exposure to VW light for 60 s or UV light for 20 s. After crosslinking, each cell-laden GelMA–RF or GelMA–IR hydrogel was transferred into DMEM or osteogenic differentiating medium for 7 days.

### 4.7. qPCR Analysis

Total RNA was isolated from each cell-laden GelMA–RF or GelMA–IR hydrogel using ISOGEN (Nippon Gene, Tokyo, Japan), following culture for 7 days [34]. cDNAs were synthesized from 1 μg of total RNA using Revetra Ace qPCR Master Mix (Toyobo, Osaka, Japan). qPCR was performed with TaqMan Universal Master Mix II (Applied Biosystems, Foster City, CA, USA) using the StepOne Real-Time PCR System (Applied Biosystems, Foster City, CA, USA). TaqMan primers (Runt-Related Transcription Factor 2 (Runx2), Mm00501584_m1; Osterix (Osx), Mm04209856_m1; Bone Sialoprotein (Bsp), Mm00492555_m1; Osteocalcin (Ocn), Mm03413826_mH) were purchased from Life Technology, Tokyo, Japan. The expression of each gene was normalized to that of 18S rRNA. The fold change between mRNA expression levels was determined as follows: fold change =2^−ΔΔCt^, where ΔΔCt = (Ct_target_ − Ct_18S rRNA_) treated group − (Ct_target_ − Ct_18S rRNA_) control group (Ct, cycle threshold).

### 4.8. Statistical Analysis

Statistical analyses were performed using the latest version of SPSS software for Windows (SPSS Inc, Chicago, IL, USA). Data are expressed as the mean ± standard deviation. Student’s *t*-test was used for comparisons. Significance was accepted at *p* < 0.05.

## 5. Conclusions

GelMA–RF hydrogel could be very useful as a graft material for bone tissue regeneration with cell transplantation.

## Abbreviations

GelMA	Gelatin methacryloyl
UV	Ultraviolet
VW	Visible wavelength
RF	Riboflavin
MA	Methacrylic anhydride
IR	Irgacure2959
Runx2	Runt-Related Transcription Factor 2
Osx	Osterix
Bsp	Bone Sialoprotein
Ocn	Osteocalcin

## References

[1] Langer R., Vacanti J.P. (1993). Tissue engineering. Science.

[2] Ikada Y. (2006). Challenges in tissue engineering. J. R. Soc. Interface.

[3] Nichol J.W., Koshy S.T., Bae H., Hwang C.M., Yamanlar S., Khademhosseini A. (2010). Cell-laden microengineered gelatin methacrylate hydrogels. Biomaterials.

[4] Noshadi I., Hong S., Sullivan K.E., Shirzaei Sani E., Portillo-Lara R., Tamayol A., Shin S.R., Gao A.E., Stoppel W.L., Black L.D. (2017). In vitro and in vivo analysis of visible light crosslinkable gelatin methacryloyl (GelMA) hydrogels. Biomater. Sci..

[5] Paul A., Manoharan V., Krafft D., Assmann A., Uquillas J.A., Shin S.R., Hasan A., Hussain M.A., Memic A., Gaharwar A.K. (2016). Nanoengineered biomimetic hydrogels for guiding human stem cell osteogenesis in three dimensional microenvironments. J. Mater. Chem..

[6] McBeth C., Lauer J., Ottersbach M., Campbell J., Sharon A., Sauer-Budge A. (2016). 3D bioprinting of GelMA scaffolds triggers mineral deposition by primary human osteoblasts. Biofabrication.

[7] Yue K., Trujillo-de Santiago G., Alvarez M.M., Tamayol A., Annabi N., Khademhosseini A. (2015). Synthesis, properties, and biomedical applications of gelatin methacryloyl (GelMA) hydrogels. Biomaterials.

[8] Selimovic S., Oh J., Bae H., Dokmeci M., Khademhosseini A. (2012). Microscale Strategies for Generating Cell-Encapsulating Hydrogels. Polymers.

[9] Dua R., Ramaswamy S. (2013). Relative survivability of human osteoblasts is enhanced by 39 degrees C and ascorbic acid after exposure to photopolymerization ingredients. Cytotechnology.

[10] Arakawa C., Ng R., Tan S., Kim S., Wu B., Lee M. (2017). Photopolymerizable chitosan-collagen hydrogels for bone tissue engineering. J. Tissue Eng. Regen. Med..

[11] Covre J.L., Cristovam P.C., Loureiro R.R., Hazarbassanov R.M., Campos M., Sato E.H., Gomes J.A. (2016). The effects of riboflavin and ultraviolet light on keratocytes cultured in vitro. Arq. Bras. Oftalmol..

[12] Husain E., Naseem I. (2008). Riboflavin-mediated cellular photoinhibition of cisplatin-induced oxidative DNA breakage in mice epidermal keratinocytes. Photodermatol. Photoimmunol. Photomed..

[13] Hardwick C.C., Herivel T.R., Hernandez S.C., Ruane P.H., Goodrich R.P. (2004). Separation, identification and quantification of riboflavin and its photoproducts in blood products using high-performance liquid chromatography with fluorescence detection: A method to support pathogen reduction technology. Photochem. Photobiol..

[14] Knappe S., Stachs O., Zhivov A., Hovakimyan M., Guthoff R. (2011). Results of confocal microscopy examinations after collagen cross-linking with riboflavin and UVA light in patients with progressive keratoconus. Ophthalmologica.

[15] Orlowska M., Koutchma T., Grapperhaus M., Gallagher J., Schaefer R., Defelice C. (2012). Continuous and Pulsed Ultraviolet Light for Nonthermal Treatment of Liquid Foods. Part 1: Effects on Quality of Fructose Solution, Apple Juice, and Milk. Food Bioprocess Technol..

[16] Bahney C.S., Lujan T.J., Hsu C.W., Bottlang M., West J.L., Johnstone B. (2011). Visible light photoinitiation of mesenchymal stem cell-laden bioresponsive hydrogels. Eur. Cells Mater..

[17] Monteiro N., Thrivikraman G., Athirasala A., Tahayeri A., Franca C.M., Ferracane J.L., Bertassoni L.E. (2018). Photopolymerization of cell-laden gelatin methacryloyl hydrogels using a dental curing light for regenerative dentistry. Dent. Mater..

[18] Eguchi T., Watanabe K., Hara E.S., Ono M., Kuboki T., Calderwood S.K. (2013). OstemiR: A novel panel of microRNA biomarkers in osteoblastic and osteocytic differentiation from mesencymal stem cells. PLoS ONE.

[19] Huang X., Chen X., Chen H., Xu D., Lin C., Peng B. (2018). Rho/Rho-associated protein kinase signaling pathway-mediated downregulation of runt-related transcription factor 2 expression promotes the differentiation of dental pulp stem cells into odontoblasts. Exp. Ther. Med..

[20] Yousefi A.M., James P.F., Akbarzadeh R., Subramanian A., Flavin C., Oudadesse H. (2016). Prospect of Stem Cells in Bone Tissue Engineering: A Review. Stem Cells Int..

[21] Sheng G. (2015). The developmental basis of mesenchymal stem/stromal cells (MSCs). BMC Dev. Biol..

[22] Engler A.J., Sen S., Sweeney H.L., Discher D.E. (2006). Matrix elasticity directs stem cell lineage specification. Cell.

[23] Huebsch N., Arany P.R., Mao A.S., Shvartsman D., Ali O.A., Bencherif S.A., Rivera-Feliciano J., Mooney D.J. (2010). Harnessing traction-mediated manipulation of the cell/matrix interface to control stem-cell fate. Nat. Mater..

[24] Khan S.N., Cammisa F.P., Sandhu H.S., Diwan A.D., Girardi F.P., Lane J.M. (2005). The biology of bone grafting. J. Am. Acad. Orthop. Surg..

[25] Janssen N.G., Weijs W.L., Koole R., Rosenberg A.J., Meijer G.J. (2014). Tissue engineering strategies for alveolar cleft reconstruction: A systematic review of the literature. Clin. Oral Investig..

[26] Khademhosseini A., Langer R. (2007). Microengineered hydrogels for tissue engineering. Biomaterials.

[27] Murtuza B., Nichol J.W., Khademhosseini A. (2009). Micro- and nanoscale control of the cardiac stem cell niche for tissue fabrication. Tissue Eng..

[28] Aubin H., Nichol J.W., Hutson C.B., Bae H., Sieminski A.L., Cropek D.M., Akhyari P., Khademhosseini A. (2010). Directed 3D cell alignment and elongation in microengineered hydrogels. Biomaterials.

[29] Bertassoni L.E., Cecconi M., Manoharan V., Nikkhah M., Hjortnaes J., Cristino A.L., Barabaschi G., Demarchi D., Dokmeci M.R., Yang Y. (2014). Hydrogel bioprinted microchannel networks for vascularization of tissue engineering constructs. Lab Chip.

[30] Kirner S.V., Guldi D.M., Megiatto J.D., Schuster D.I. (2015). Synthesis and photophysical properties of new catenated electron donor-acceptor materials with magnesium and free base porphyrins as donors and C60 as the acceptor. Nanoscale.

[31] Nguyen A.K., Gittard S.D., Koroleva A., Schlie S., Gaidukeviciute A., Chichkov B.N., Narayan R.J. (2013). Two-photon polymerization of polyethylene glycol diacrylate scaffolds with riboflavin and triethanolamine used as a water-soluble photoinitiator. Regen. Med..

[32] Fairbanks B.D., Schwartz M.P., Bowman C.N., Anseth K.S. (2009). Photoinitiated polymerization of PEG-diacrylate with lithium phenyl-2,4,6-trimethylbenzoylphosphinate: Polymerization rate and cytocompatibility. Biomaterials.

[33] Matsubara T., Kida K., Yamaguchi A., Hata K., Ichida F., Meguro H., Aburatani H., Nishimura R., Yoneda T. (2008). BMP2 regulates Osterix through Msx2 and Runx2 during osteoblast differentiation. J. Biol. Chem..

[34] Nishida E., Sasaki T., Ishikawa S.K., Kosaka K., Aino M., Noguchi T., Teranaka T., Shimizu N., Saito M. (2007). Transcriptome database KK-Periome for periodontal ligament development: Expression profiles of the extracellular matrix genes. Gene.

